# Draft genome sequence of *Paenibacillus* sp. strain RC67, an isolate from a long-term forest soil warming experiment in Petersham, Massachusetts

**DOI:** 10.1128/MRA.00373-23

**Published:** 2023-10-12

**Authors:** Wyatt C. Tran, Brendan Sullivan, Claire E. Kitzmiller, Mallory Choudoir, Rachel Simoes, Nipuni Dayarathne, Kristen M. DeAngelis

**Affiliations:** 1 Department of Microbiology, University of Massachusetts, Amherst, Massachusetts, USA; 2 Department of Plant and Microbial Biology, North Carolina State University, Raleigh, North Carolina, USA; Indiana University, Bloomington, Indiana

**Keywords:** soil microbiology, microbial ecology, bioinformatics

## Abstract

*Paenibacillus* sp. strain RC67 was isolated from the Harvard Forest long-term soil warming experiment. The assembled genome is a single contig with 7,963,753 bp and 99.4% completion. Genome annotation suggests that the isolate is of a novel bacterial species.

## ANNOUNCEMENT

Soil microbes mediate nutrient cycling, but it remains elusive how climate warming impacts microorganisms and their metabolism. The ongoing Harvard Forest soil warming experiment investigates the influence of warming temperature on soils (1). *Paenibacillus* sp. strain RC67 was isolated from the Harvard Forest in Petersham, Massachusetts and sequenced to understand the impact of warmer climate on soil bacterial genomes. The genome sequence indicates that this isolate is a novel bacterial species belonging to the *Paenibacillus* genus.

RC67 was isolated from 1 g of mineral soil collected 10 cm below surface at an elevation of 355 m with a steel corer in 2022 from a heated plot (43°N, 72.18°W) using ISP2 (2) medium in aerobic conditions. For gDNA extraction, RC67 was grown on 10% tryptic soy broth (TSB) at 30°C with shaking at 150 rpm until an OD of 0.5 was reached. Cells were pelleted using centrifugation at 4,000 rpm for 15 minutes, and genomic DNA was extracted using CTAB method (3). The library was prepared using Ligation Sequencing Kit SQK-LSK-109 from Oxford Nanopore Technologies (4). The DNA was not sheared or size selected. The genome was sequenced using Oxford Nanopore sequencing technology at SeqCenter (Pittsburgh, PA). R9.4.1 flowcells were run on GridION platform, and Guppy v4.5.5 was used for high-accuracy basecalling to archive Q20 performance and 288,137,203 bp.

The genome was assembled, annotated, and analyzed as part of the Bioinformatics Lab (MICROBIO 590B) course at the University of Massachusetts Amherst (5). Default parameters were used for all software unless otherwise specified. To estimate the genome size, the 16S rRNA gene was sequenced (3), and BLAST (6) determined that the closest related organism with an available genome is *Paenibacillus rigui* (accession number: NR_116517 [97.03% similarity]) with a 7.173-Mb genome size. Filtlong v0.2.1 (7) filtered 85% of the highest quality reads with minimum length of 1,000 bp to target 40× coverage, which yielded 577,588,430 bp. *De novo* assembly was performed using Flye v2.8.1 (8). A consensus assembly was generated using Minimap2 v2.17 (9) and Racon v1.4.3 (10), followed by a final polishing using Medaka v1.5.0 (11). The genome was not trimmed, rotated, or circularized.

**Fig 1:**
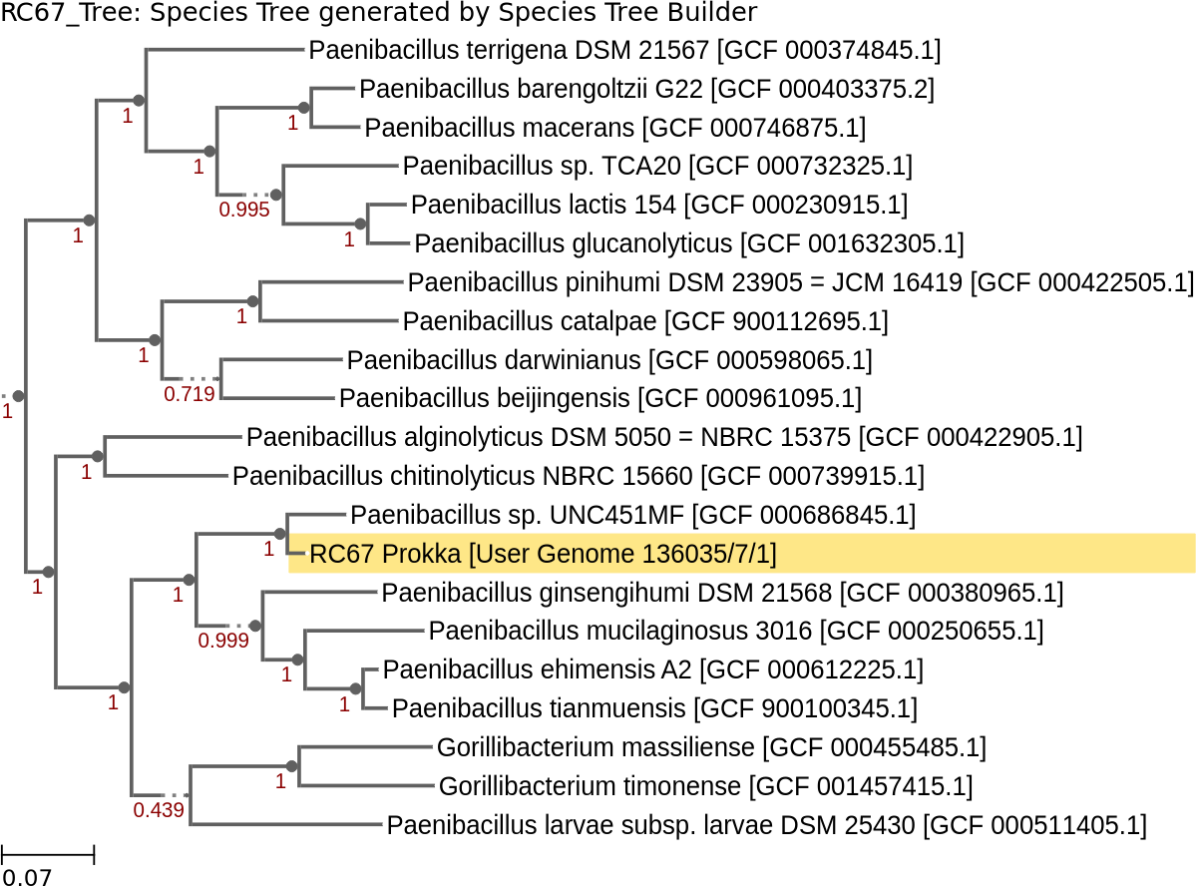
Phylogenetic tree constructed through estimating the approximate maximum likelihood of phylogeny from the concatenated multiple sequence alignments (MSAs). The phylogenetic tree was generated based on default parameters on the Insert Genome Into Species Tree v2.2.0 (20), which uses MSAs for each 49 core universal genes defined by Clusters of Orthologous Groups, and relatedness is determined by alignment similarity.

The RC67-assembled genome was uploaded to KBase (12) for annotation, and quality was assessed using QUAST v4.4 (13). The RC67 genome was annotated using Prokka v1.14.5 (14). The genome assembled into a single contig with an N50 of 7,963,753 bp. CheckM v1.018 (15) indicated a completion of 99.4% and a contamination of 2.07%. Prokka annotation indicated the presence of 23S, 16S, and 5S rRNA genes with 99 tRNA genes for 38 tRNAs, a high-quality assembly (16). Classify microbes with GTDB-Tk v1.7.0 (17) matched RC67 to the Bacteria domain, Bacillus phylum, Bacilli class, Bacillales order, Paenibacillaceae family, and *Paenibacillus* genus. The closest sequenced genome from the RefSeq database to RC67 was an unclassified *Paenibacillus* sp. UNC451MF (Fig. 1). FastANI v.0.1.3 (18, 19) determined the ANI of RC67 to *Paenibacillus* sp. UNC451MF to be 85.91%. The novel isolate may provide a further understanding on the impact of warming climate on *Paenibacillus*.

## Data Availability

The 16S rRNA gene sequence accession number is OQ581834. The raw whole-genome sequence reads are available in GenBank under the BioProject accession number PRJNA949990. The Sequence Read Archive (SRA) accession number is SRR24091469 with a BioSample accession number of SAMN33969015. Public KBase narrative of *Paenibacillus* sp. strain RC67 genome annotation is available on Kbase under the title “Draft Genome Sequence of Paenibacillus sp. strain iRC67, an Isolate from a Long-Term Forest Soil Warming Experiment in Petersham, Massachusetts.”
